# Effects of antibacterial mineral leachates on the cellular ultrastructure, morphology, and membrane integrity of *Escherichia coli *and methicillin-resistant *Staphylococcus aureus*

**DOI:** 10.1186/1476-0711-9-26

**Published:** 2010-09-16

**Authors:** Caitlin C Otto, Tanya M Cunningham, Michael R Hansen, Shelley E Haydel

**Affiliations:** 1 School of Life Sciences, Arizona State University, Tempe, AZ, USA; 2 The Biodesign Institute Center for Infectious Diseases and Vaccinology, Arizona State University, Tempe, AZ, USA

## Abstract

**Background:**

We have previously identified two mineral mixtures, CB07 and BY07, and their respective aqueous leachates that exhibit *in vitro *antibacterial activity against a broad spectrum of pathogens. The present study assesses cellular ultrastructure and membrane integrity of methicillin-resistant *Staphylococcus aureus *(MRSA) and *Escherichia coli *after exposure to CB07 and BY07 aqueous leachates.

**Methods:**

We used scanning and transmission electron microscopy to evaluate *E. coli *and MRSA ultrastructure and morphology following exposure to antibacterial leachates. Additionally, we employed *Bac*light LIVE/DEAD staining and flow cytometry to investigate the cellular membrane as a possible target for antibacterial activity.

**Results:**

Scanning electron microscopy (SEM) and transmission electron microscopy (TEM) imaging of *E. coli *and MRSA revealed intact cells following exposure to antibacterial mineral leachates. TEM images of MRSA showed disruption of the cytoplasmic contents, distorted cell shape, irregular membranes, and distorted septa of dividing cells. TEM images of *E. coli *exposed to leachates exhibited different patterns of cytoplasmic condensation with respect to the controls and no apparent change in cell envelope structure. Although bactericidal activity of the leachates occurs more rapidly in *E. coli *than in MRSA, LIVE/DEAD staining demonstrated that the membrane of *E. coli *remains intact, while the MRSA membrane is permeabilized following exposure to the leachates.

**Conclusions:**

These data suggest that the leachate antibacterial mechanism of action differs for Gram-positive and Gram-negative organisms. Upon antibacterial mineral leachate exposure, structural integrity is retained, however, compromised membrane integrity accounts for bactericidal activity in Gram-positive, but not in Gram-negative cells.

## Background

With the advent of antibiotics in the early 20^th ^century, morbidity and mortality from bacterial infections were dramatically reduced in the industrialized world. In recent decades, these advances have been tempered by the rapid, widespread emergence of microorganisms that are resistant to multiple, commonly used antibiotics [1]. As our arsenal of effective antibiotics is diminishing, the pursuit of novel therapeutic agents is becoming progressively more urgent.

Minerals have been utilized in traditional medicine for centuries as topical treatments for cutaneous wounds, digestive treatments for gastrointestinal ailments, nutritional supplements, and for removal of toxins from the body [2-4]. Traditionally, the mechanism of mineral-based healing activities has been attributed to physical properties, such as the expansive surface area and resulting highly adsorptive properties of clays present in the mixtures [2].

Recently, various mineral products marketed for their health benefits have been investigated for their potential antimicrobial properties [5-8]. However, only a small number of clay products have been shown to be antibacterial and the mechanism of antibacterial activity has been elucidated for very few of these products [8]. Falkinham et al. [8] attributed the antibacterial effects of Jordan's red soils to bacteriocins produced by bacteria present in the clays. It was hypothesized that application of the red soil to an infected area of the skin allowed the inherent organisms to proliferate, produce bacteriocins, and thus kill the infectious pathogens [8]. Mpuchane et al. [7,9] tested a total of 102 clays from South Africa and determined that only nine of these clay samples had antibacterial activity. The antibacterial properties of these South African medicinal clays were attributed to the low pH environment of the hydrated mineral suspensions (pH < 4), and it was further postulated that metal cations could contribute to toxicity [7,9]. While Mpuchane et al. [9] determined that nine clays had antibacterial properties, none of the clays specifically sold for use against bacterial infections had antibacterial activity. Therefore, it is essential to scientifically validate the efficacy of these mineral products prior to use in a clinical setting.

Clay minerals are excellent adsorbent materials due to their small particle size (< 2 μm), stable layered structure, and high cation exchange capacity [10]. In a pH-dependent manner, exchangeable cations can bind to the clay surface, balancing the negative charge of the clay structure. In hydrated suspensions, the adsorbate can then be released into the aqueous solution, varying the cationic composition of the solution [10,11]. These released metal ions are known to have toxic effects on bacteria by competing with essential enzyme cofactors, irreversibly binding biological molecules to inhibit function, replacing ions essential to membrane stabilization, and inducing DNA mutations [12-15]. For example, metal cations, such as iron, copper, and chromium, have been implicated in production of elevated levels of reactive oxygen species which can lead to DNA damage, lipid peroxidation, protein oxidation, and eventual cell death [16-18]. Metal ion toxicity varies with pH and appears to be related to changes in ion species that occur as the pH is adjusted [12,15,19]. These alterations in toxicity are due to the relative abilities of the ion species to bind cell surfaces and exert their effects [12].

In a prior study, we identified two mineral mixtures, arbitrarily designated BY07 and CB07, that exhibit antibacterial activity [5]. From these mineral mixtures, we prepared aqueous leachates that contain metal ions released from the clay minerals, but are absent of all solid particles. These leachates retain antibacterial activity, establishing that the mechanism of action is dependent on chemical, not physical interactions [5]. Further investigations revealed that the antibacterial activity of BY07 and CB07 mineral mixtures is related to the pH-dependent bioavailability of toxic metal ions in a low pH environment [5]. While we have discovered that pH-dependent ion toxicity mediates CB07 and BY07 antibacterial activity, further investigations must be performed to fully understand the precise mechanism of action. In this study, we assessed whether cell lysis occurs in *E. coli *and MRSA cells during leachate exposure and investigated cellular membrane integrity as possible mechanisms of action of the aqueous leachates.

## Methods

### Bacterial strains and growth conditions

*E. coli *ATCC 25922, obtained from the American Type Culture Collection, and MRSA, obtained from Sonora Quest Laboratories (Tempe, AZ, USA), were used for all studies as previously described [6]. *E. coli *was grown on Luria-Bertani (LB) agar or in LB broth, and MRSA was grown on trypticase soy agar (TSA) or in trypticase soy broth (TSB). Both bacterial strains were grown at 37°C with gentle rotary mixing.

### Mineral leachate preparation

Mineralogical and major chemical characterization of the CB07 and BY07 mineral mixtures has been previously described [5]. Briefly, the CB07 mineral is primarily composed of quartz (45.5%), illite (19.8%), and calcium smectite (17.2%), while the BY07 mineral primarily consists of calcium smectite (37.3%), anorthoclase feldspar (23.0%), and quartz (13.7%) [5]. Major oxide chemical analyses reveal that CB07 and BY07 are primarily composed of silicon, aluminum, iron, calcium, sodium, potassium, and sulfur [5]. Leachates of CB07 and BY07 mineral samples were prepared as previously described [5]. Briefly, 1 g of autoclaved minerals was vigorously agitated in 20 mL of UV-irradiated, ultrapure, deionized H_2_O (dH_2_O) for 18 - 24 hours at room temperature. The suspension was centrifuged at 31,000 × g for 3 h to remove the remaining insoluble minerals and then sterilized by passage through a 0.22 μm filter.

### Antibacterial susceptibility testing of mineral leachates

*E. coli *and MRSA exponential phase cultures were prepared by diluting overnight cultures into fresh growth medium to a concentration of 10^7 ^CFU/mL and continuing growth at 37°C with gentle rotary mixing until the cultures reached mid-logarithmic phase of growth. Bacterial cells were collected by centrifugation, washed once in phosphate-buffered saline (PBS), and suspended in the appropriate leachate solution or sterile dH_2_O at an initial concentration of 10^7 ^CFU/mL. Initial CFU concentrations were confirmed by plating the control bacterial population and enumerating colonies after 24 h incubation at 37°C. Due to experimental sample processing, the 0 h experimental exposure times were ~ 3 min. Experimental samples were incubated at 37°C with gentle rotary mixing for a specified time, and cell survival was determined by plating duplicate 10-fold serial dilutions for each sample at appropriate time points and enumerating colonies after 24 h incubation at 37°C.

### Scanning electron microscopy (SEM)

Bacterial cells were prepared for SEM as described above, with the exceptions that cultures were collected at late logarithmic phase of growth and initial concentrations were 10^8 ^CFU/ml. Following 24 h exposure to the leachates, washed cells were inoculated onto a poly-L-lysine-coated coverglass slide and allowed to adhere for 5 min at room temperature. After washing the slide in 50 mM sodium phosphate buffer, pH 7, the cells were chemically crosslinked onto the slide in 2% gluteraldehyde (buffered in 50 mM sodium phosphate, pH 7). The immobilized cells were then fixed in 2% osmium tetroxide for 15 min at room temperature, washed three times in 50 mM sodium phosphate buffer, and dehydrated in 5 min washes in a sequential acetone series (20%, 40%, 60%, 80%, 3× 100%). The samples were critical point dried in a Balzers 020 critical point dryer, attached to aluminum mounting stubs, sputter coated with gold-palladium, and imaged with an XL30 Environmental SEM equipped with a field emission gun. A minimum of 200 cells was counted from each of three independent replicates.

### Transmission electron microscopy (TEM)

*E. coli *and MRSA exponential phase cultures were prepared as described above for SEM with an initial concentration of 10^8 ^CFU/mL. Following 24 h exposure to the leachates, cells were fixed in 2% gluteraldehyde buffered in 50 mM phosphate, pH 7, for 2 h at room temperature. The cells were then washed in 50 mM phosphate and resuspended in 1% agarose (final concentration). The agarose-embedded cell pellets were fixed in 2% osmium tetroxide (buffered in 50 mM phosphate) for 2 h at room temperature, washed three times in 50 mM phosphate buffer, washed three times in dH_2_O, and en bloc stained in 0.5% uranyl acetate overnight at 4°C. The pellets were dehydrated in 10 min washes with a sequential acetone series (20%, 40%, 60%, 80%, 3× 100%) and infiltrated with Spurr's resin. Thin sections (70 nm) were cut using an Ultracut R ultramicrotome (Leica Microsystems, Vienna, Austria). Sections were captured on formvar-coated, 300-mesh copper grids, post-stained in uranyl acetate and Sato's lead citrate, and observed on a Philips CM12 TEM at 80 kV. A minimum of 60 cells was counted from each of three independent replicates.

### Flow cytometric measurements

To evaluate the membrane integrity of *E. coli *and MRSA following exposure to the leachates, the *Bac*Light LIVE/DEAD membrane permeability kit (Invitrogen, Carlsbad, CA, USA) was used following the manufacturer guidelines. *E. coli *and MRSA mid-logartithmic phase cultures were prepared as described above and harvested at an initial concentration of 10^8 ^CFU/mL. A standard curve was prepared by mixing live (0.85% saline-exposed) cells and dead (40% isopropanol-exposed) cells together at various proportions of live:dead cells (100%, 75%, 50%, 25%, 0% alive). Following exposure to the leachates or control conditions, cells were incubated in 5 μM SYTO9 and 30 μM propidium iodide (PI) for 15 min in the dark and then immediately subjected to flow cytometric analysis. *E. coli *cells were analyzed following 1 h exposure to CB07 leachate (CB07-L) and 6 h exposure to BY07 leachate (BY07-L), while MRSA cells were analyzed following 15 h exposure to either CB07-L or BY07-L. These time points represent the exposure time required for bactericidal activity (≥ 99.9% killing) of the different leachates against the two cell types. A Cytomics FC 500 flow cytometer (Beckman Coulter, Inc., Brea, CA, USA) fitted with a 488 nm excitation laser was used for membrane permeability analyses. Green fluorescence was detected on channel FL1 with a 525 nm bandpass filter. Red fluorescence was detected on channel FL3 with a 620 bandpass filter. Since the SYTO9 dye emits a strong signal at a wavelength of 600 nm, it overlaps with the PI emission [20]. Therefore, membrane permeabilization is determined by a horizontal population shift that occurs down the green fluorescent intensity axis. For each series of flow cytometric measurements, 50,000 cells were counted and analyzed.

## Results

### Antibacterial mineral leachates

**Figure 1:**
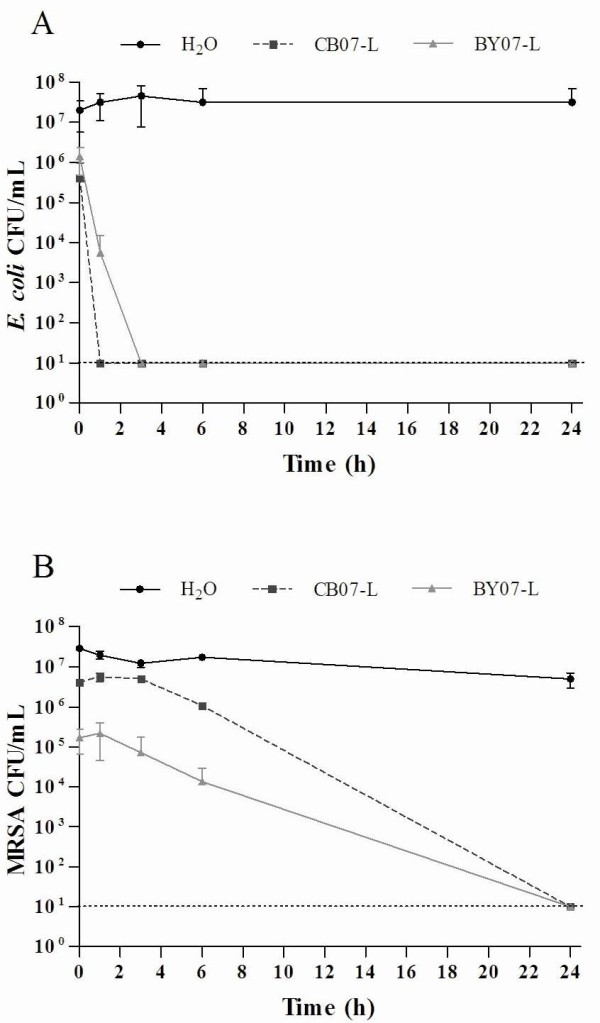
***E. coli *(a) and MRSA (b) survival in CB07 and BY07 mineral leachates for 24 h**. Values represent the mean CFU and SD of at least three independent experiments. The dotted line represents the limit of CFU detection.

The effects of BY07-L and CB07-L on the growth of *E. coli *and MRSA were investigated by performing *in vitro *antimicrobial susceptibility experiments. Bacteria, at initial concentrations of 10^7 ^CFU/mL, were incubated in BY07-L or CB07-L for 24 h prior to plating to determine viability. Due to variable bactericidal activity of the leachates, it is essential to regularly test the antibacterial activity of the leachates. For example, we previously demonstrated that *E. coli *is completely killed by BY07-L after 18 h [5]. However, in the present study, exposure to BY07-L completely killed *E. coli *after a 3 h incubation (Figure 1a). *E. coli *viability was completely eliminated following 24 h exposure to both mineral leachates (Figure 1a). At the 0 h time point, leachate-exposed *E. coli *and MRSA cell viability was consistently 1-log_10 _unit lower than the respective dH_2_O controls (Figure 1). Experimental processing, requiring approximately 3 min to complete, accounted for the initial decrease in cell viability and also demonstrated the rapid bactericidal activity of the two leachates. With respect to the 6 h water control, MRSA exposure to CB07-L resulted in a > 1-log_10 _unit reduction in viability after 6 h (Figure 1b). Alternatively, exposure to BY07-L for 6 h resulted in bactericidal activity (Figure 1b). In both cases, MRSA viability was completely eliminated after 24 h (Figure 1b).

### Scanning electron microscopy

**Figure 2:**
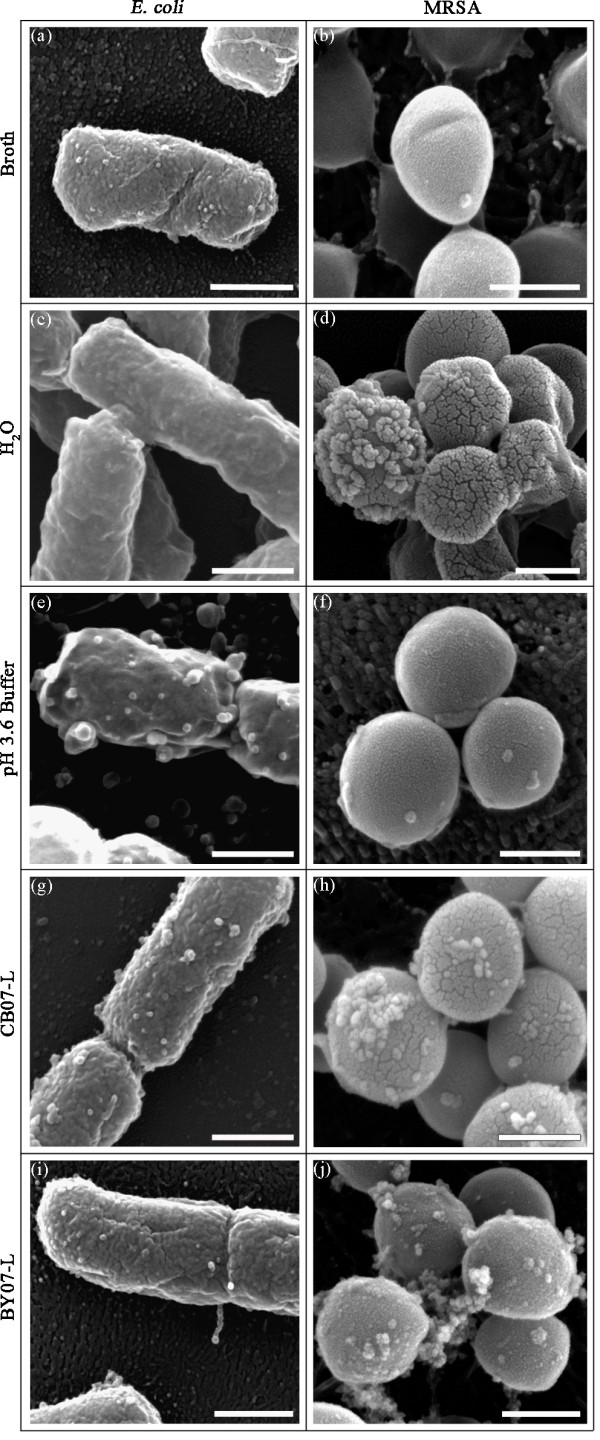
**SEM images of *E. coli *(a, c, e, g, i) and MRSA (b, d, f, h, j) after 24 h incubation in broth, dH_2_O, pH 3.6 buffer, CB07-L, or BY07-L**. Scale bar = 500 nm.

**Figure 3:**
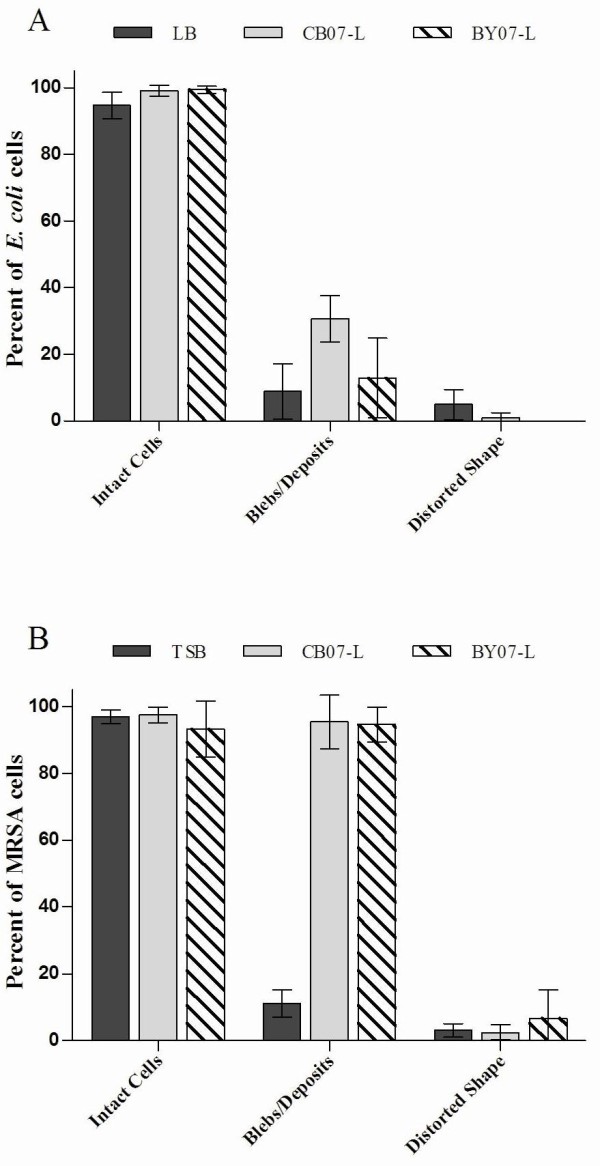
**Frequency of characteristics observed in three independent replicates of SEM images of *E. coli *(a) and MRSA (b)**. For each independent replicate, at least 200 cells were visualized and scored for the observed characteristic.

SEM and TEM were used to directly observe morphological and ultrastructural changes induced in *E. coli *and MRSA upon exposure to and killing by the BY07 and CB07 antibacterial leachates. As shown in Figure 1a, *E. coli *was completely killed after 24 h exposure to BY07-L and CB07-L. SEM images of *E. coli *showed that the cells did not lyse following exposure to the leachates (Figures 2g and 2i). Enumeration of imaged cells revealed that > 99.0% of leachate-exposed cells remain intact and that damaged or lysed cells were only observed in < 1% of CB07-L- and BY07-L-exposed cells (Figure 3a). SEM images of *E. coli *cells grown in LB broth showed a rough cell surface with discrete ridges (Figure 2a). Leachate-treated (Figures 2g and 2i) *E. coli *cells also exhibited a rough cell surface with discrete ridges, while water-incubated (Figure 2c) and low pH buffer-treated (Figure 2e) *E. coli *cells had a wavy and smooth cell surface appearance. Further, 30.7% of CB07-L-treated cells exhibited the appearance of membrane bleb-like structures or deposits, while 12.9% of BY07-L-treated cells had apparent blebs or deposits on the cell surface (Figure 3a). Overall, both CB07-L- and BY07-L-treated *E. coli *cells maintained their rod shape with very few distorted cells (Figure 3a).

As shown in Figure 1b, MRSA was completely killed after 24 h exposure to the leachates. SEM images of MRSA demonstrated intact cells following exposure to CB07-L and BY07-L (Figures 2h and 2j), with damaged or lysed cells observed in 2.5% and 6.8% of cells, respectively (Figure 3b). MRSA cells incubated in water for 24 h showed flattened and distorted cells with bleb-like structures or deposits (Figure 2d), while cells in broth and low pH buffer exhibited a smooth cell surface appearance (Figures 2b and 2f). In contrast, following exposure to the leachates, the MRSA cell surface appeared rough, showed the appearance of bleb-like structures, and had an increased abundance of extracellular debris (Figures 2h and 2j). Further, 95.4% and 94.5%, respectively, of CB07-L- and BY07-L-treated cells exhibited bleb-like structures, while only 11.1% of cells grown in TSB showed blebs (Figure 3b).

### Transmission electron microscopy

**Figure 4:**
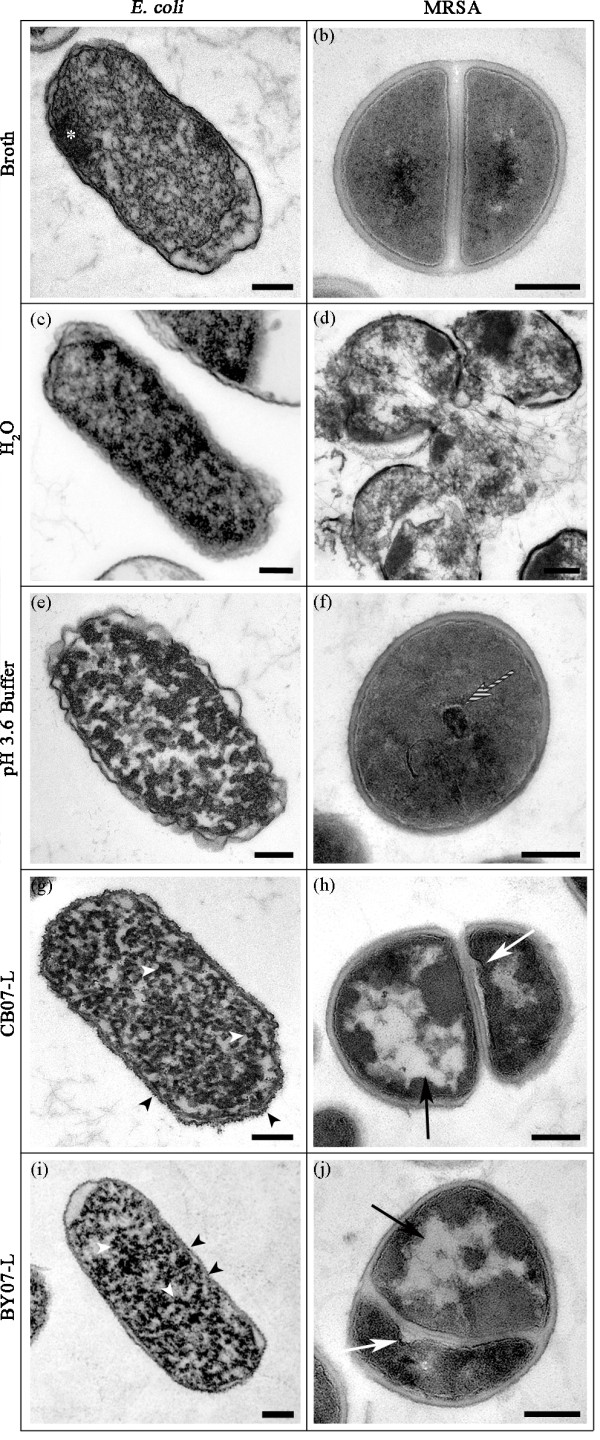
**TEM images of *E. coli *(a, c, e, g, i) and MRSA (b, d, f, h, j) after 24 h incubation in broth, dH_2_O, pH 3.6 buffer, CB07-L, or BY07-L**. Scale bar = 200 nm. Electron-dense region, asterisk (a); mesosome-like structure, striped arrow (f); granules, black arrowheads (g, i); cytoplasmic condensation, white arrowheads (g, i); cytoplasmic disruption, black arrows (h, j); cytoplasmic membrane disruptions, white arrows (h, j).

**Figure 5:**
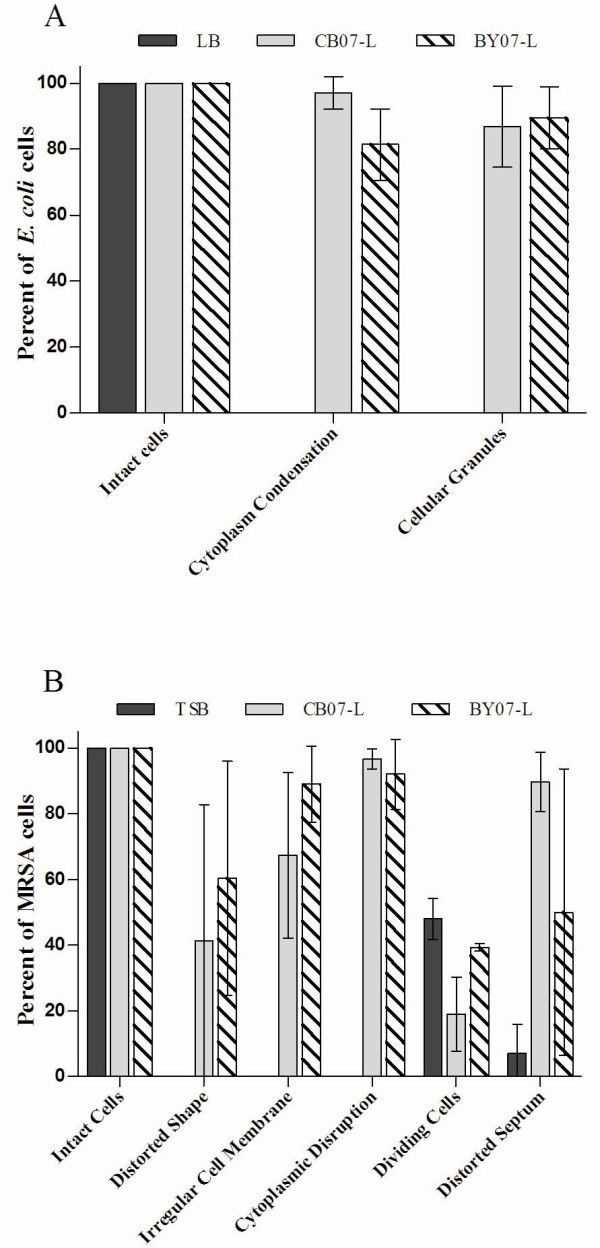
**(a) Frequency of characteristics observed in three independent replicates of TEM images of *E. coli *(a) and MRSA (b)**. For each independent replicate, at least 60 cells were visualized and scored for the observed characteristic.

TEM images of *E. coli *further confirmed that the cells remain intact following exposure to the leachates (Figures 4g,i, and 5a), indicating that cell lysis is not the antibacterial mechanism of action. TEM images of *E. coli *grown in LB for 24 h showed electron-dense regions (Figure 4a; white asterisk) and evidence of cytoplasmic condensation (Figure 4a). The electron-dense regions are characteristic of *E. coli *cells grown into stationary phase and are likely due to the accumulation of glycogen inclusion bodies [21]. *E. coli *exposed to CB07-L and BY07-L displayed condensation of the cytoplasmic contents (Figures 4g and 4i; white arrowheads) in 97.0% and 81.4% of cells, respectively (Figure 5a). Additionally, small (diameter of ~ 10 nm) electron-dense, deposited or cellular-based granules bound to the cell envelope were observed (Figures 4g and 4i; black arrowheads). These granules were observed in 86.8% and 89.4% of cells exposed to CB07-L and BY07-L, respectively (Figure 5a). Feng et al. [22] reported similar electron-dense granules in TEM following *E. coli *and MRSA exposure to silver ions, hypothesizing that silver toxicity is due to ions entering the cells and binding to sulfhydryl groups [22]. Because the mineral leachates were generated using water [5], both *E. coli *and MRSA cells were exposed to dH_2_O to resolve the effects of prolonged water incubations on cellular viability. Following 24 h exposure to dH_2_O, *E. coli *viability was not significantly affected (Figures 1a and 1b). As evident in the TEM images, the effects of extended exposure of *E. coli *to dH_2_O were unremarkable, bearing similarity to the LB-exposed cells (Figures 4a and 4c). Non-uniform spacing between the *E. coli *cytoplasmic contents and cell envelope was observed in water-treated, broth-treated, and BY07-L- and CB07-L-treated cells (Figures 4a,c,g, and 4i).

TEM images of MRSA confirmed that the cells remain intact following exposure to the leachates (Figures 4h and 4j), indicating that cell lysis is not the antibacterial mechanism of action in Gram-positive cells. Enumeration of triplicate TEM sample images verified that 100% of leachate-exposed cells remain intact (Figure 5b). Cytoplasmic disruption of MRSA (Figure 4h and 4j; black arrows) following exposure to CB07-L and BY07-L was observed in 96.7% and 92% of cells, respectively (Figure 5b). Possible disruptions in the cytoplasmic membrane (Figures 4h and 4j; white arrows) were observed in 67.3% and 89.0% of CB07-L- and BY07-L-treated cells, respectively (Figure 5b). Cells with a distorted shape were also observed in triplicate samples of MRSA exposed to the mineral leachates. When compared to the broth control, a decreased frequency of dividing cells was observed in leachate-exposed MRSA cells (Figure 5b). Moreover, many of these leachate-exposed dividing cells showed evidence of a distorted septum (Figure 5b). Following 24 h exposure to dH_2_O, MRSA viability decreased by 1-log_10 _unit (Figure 1b). Water-exposed MRSA cells exhibited evidence of hypo-osmotic environmental stress through cell lysis, wavy cell envelope structures, and separation of cytoplasmic contents from the membrane (Figure 4d). Notably, these ultrastructural alterations were not evident in the broth-exposed or leachate-exposed cells (Figures 4b,h, and 4j), thus indicating that leachate-induced toxicity differs significantly from prolonged cell incubation in water.

CB07-L and BY07-L generate low pH environments ranging between 3.3 - 3.7 [5]. To evaluate the effects of the low pH on cellular ultrastructure, we exposed *E. coli *and MRSA to a 100 mM phosphate buffer at pH 3.6 to mimic the low pH environment of the leachates. After 24 h exposure to low pH buffer, *E. coli *viability was reduced by 3-log_10 _units [5] as compared to the complete loss of viability observed after 24 h exposure to CB07-L and BY07-L (Figure 1a). This maintenance of viability was expected since *E. coli *exhibits an inducible acid tolerance in order to facilitate passage through the low pH environment of the digestive tract [23]. Low pH buffer-exposed *E. coli *displayed different patterns of cytoplasmic condensation from that of the leachate-exposed cells (Figures 4e,g, and 4i). In contrast to *E. coli*, MRSA cells exposed to the low pH buffer for 24 h were killed completely (data not shown). TEM images of low pH buffer-exposed MRSA cells revealed an even distribution of cytoplasmic contents (Figure 4f), similar to cells grown in broth (Figure 4b), demonstrating that the toxic effects induced by a low pH environment differ from that of the antibacterial leachates. Images of low pH buffer-exposed MRSA cells exhibited mesosome-like structures (Figure 4f; striped arrow), however, this effect was only observed in the low pH buffer-exposed cells and was likely a processing artifact due to exposure to the low pH phosphate buffer.

### Membrane Permeability

While electron microscopy (EM) provides useful insight into the mechanism of action of antibacterial agents, the resulting images are observational only. Other techniques must, therefore, be used in tandem to verify the observations generated from the EM images. Accordingly, we investigated the effects of BY07-L and CB07-L on the membrane permeability of *E. coli *and MRSA by using the *Bac*Light LIVE/DEAD bacterial viability kit. This assay uses two DNA intercalating dyes: green fluorescent SYTO9, which penetrates all membranes and red fluorescent propidium iodide (PI), which can only penetrate permeabilized membranes due to its large size and negative charge [24]. Red fluorescence is produced in the membrane-permeabilized cell by combined displacement of SYTO9 by PI and quenching of SYTO9 emission by fluorescence resonance energy transfer (FRET) [20].

**Figure 6:**
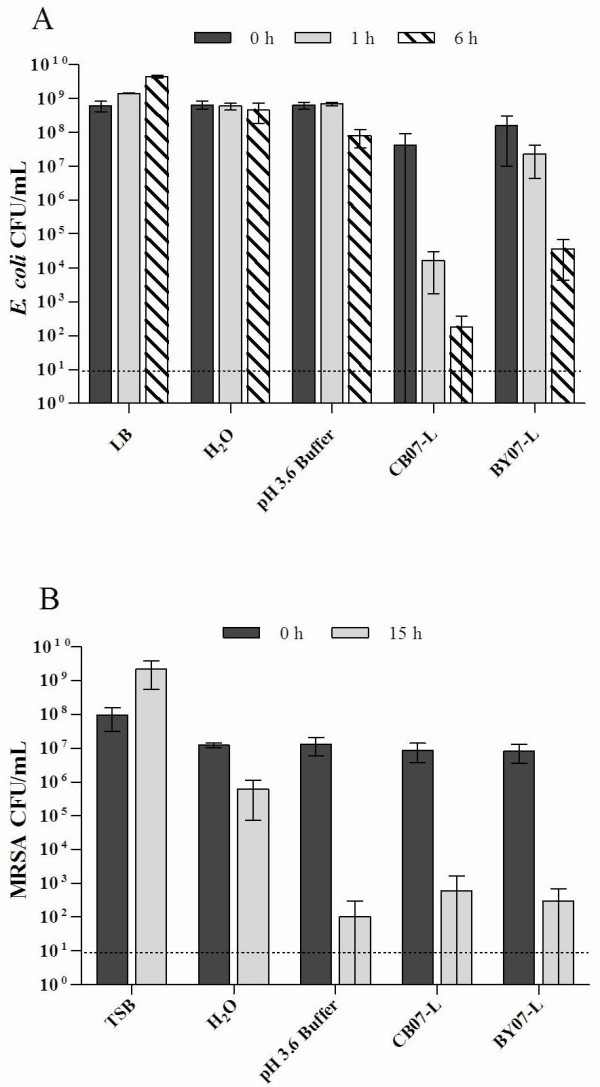
***E. coli *(a) viability determined by CFU plate counts following 1 h exposure to CB07-L and BY07-L**. MRSA (a) viability determined by CFU plate counts following 15 h exposure to mineral leachates. The dotted line represents the limit of CFU detection.

CB07-L and BY07-L rapidly kill higher concentrations of *E. coli*, with bactericidal activity occurring after 1 h and 6 h, respectively (Figure 6a). Because membrane permeabilization can naturally occur following cell death, these time points were used as the minimum period of time required for bactericidal activity to directly evaluate the effects of the antibacterial leachates on *E. coli *membrane integrity.

**Figure 7:**
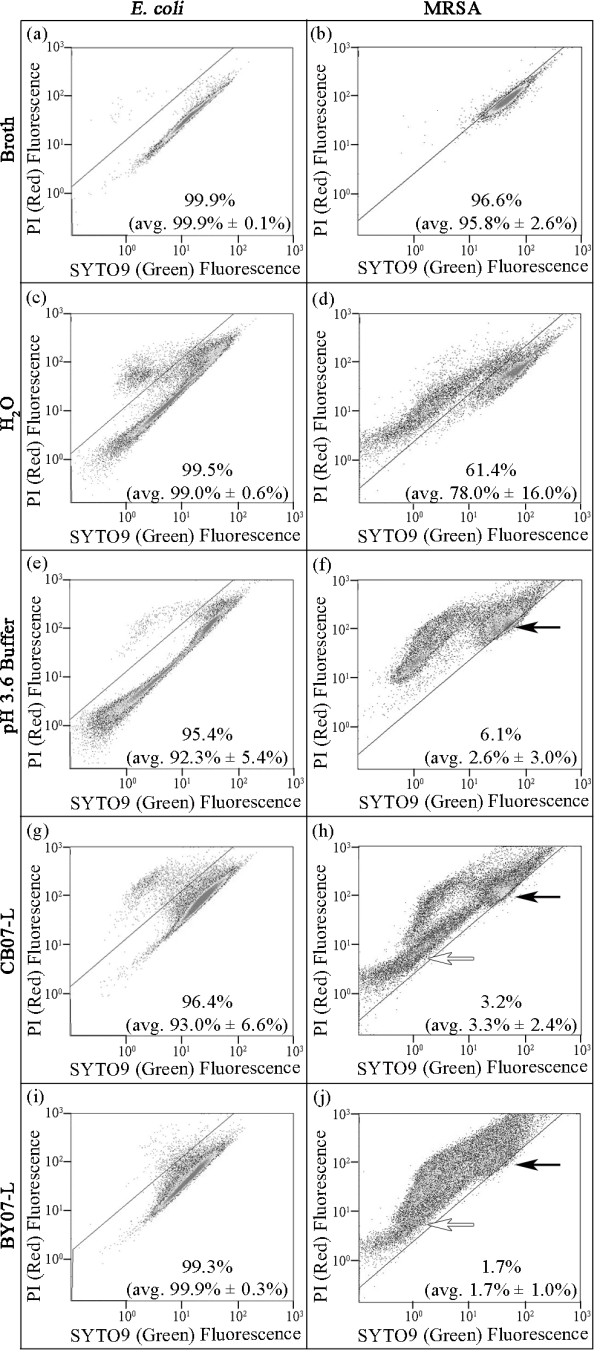
**Flow cytometric analysis of *E. coli *(a, c, e, g, i) and MRSA (b, d, f, h, j) after incubation in broth, dH_2_O, pH 3.6 buffer, CB07-L, or BY07-L**. Two red fluorescent populations were observed with a higher fluorescence intensity (black arrow) and lower fluorescence intensity (white arrow). Flow cytometric analyses and percentages were derived from a single representative experiment. Average values from at least three independent replicate experiments are reported in parentheses. Percentages denote the number of cells that remained impermeable to PI, and therefore fluoresced green. Green fluorescence was detected on channel FL1 with a 525 nm ± 10 nm bandpass filter for SYTO9; red fluorescence was detected on channel FL3 with a ≥ 620 bandpass filter for PI.

Following 1 h incubation in LB broth, *E. coli *viability was assessed by CFU enumeration on agar plates and resulted in an average of 10^9 ^CFU/mL (Figure 6a). Given that *E. coli *remains viable in broth, flow cytometric analysis resulted in an average of 99.9% of cells with intact membranes (Figure 7a; Table 1). Although 1 h exposure to CB07-L elicited bactericidal activity (Figure 6a), flow cytometry revealed that 93.0% (average) of *E. coli *cells remained impermeable to PI (Figure 7g; Table 1). Similarly, flow cytometry revealed that 99.3% of *E. coli *cells exposed to BY07-L for 6 h were impermeable to PI (Figure 7i; Table 1). These data were confirmed in triplicate flow cytometric experiments (Figure 7; Table 1) and indicate that membrane permeability does not occur in *E. coli *following exposure to the leachates, and thus, is not the primary mechanism of action for either of the mineral leachates (Figure 7; Table 1). *E. coli *cells were also exposed to a low pH buffer control to mimic the low pH environment of the leachates. Minimal membrane permeability was observed after 1 h exposure to low pH buffer (Figure 7e), with an average of 92.3% impermeable cells detected by flow cytometry (Figure 7e; Table 1). Table 1 summarizes the two data collection methods, showing that flow cytometry data suggest an insignificant loss in viability, despite the bactericidal activity demonstrated by CFU enumeration (Figure 7; Table 1).

**Table 1:** Summary of *E. coli *survival and membrane permeability following incubation in LB, water, pH 3.6 phosphate buffer, and mineral leachates

Treatment	Time (h)	Flow cytometry, percent intact membranes **(avg ± SD)**^**a**^	Plate count viability, **CFU enumeration (%)**^**b**^
LB	1	99.9 (99.9 ± 0.1)	0.5-log_10 _Increase (n/a)^c^
			
dH_2_O	1	99.5 (99.0 ± 0.6)	< 0.5-log_10 _Decrease (50.0)
			
pH 3.6 Buffer	1	95.4 (92.3 ± 5.4)	< 0.5-log_10 _Decrease (50.0)
			
CB07-L	1	96.4 (93.0 ± 6.6)	1.5-log_10 _Decrease (5.0)
			
BY07-L	6	99.3 (99.9 ± 0.3)	2.5-log_10 _Decrease (0.5)

^a ^Actual percentages based on 50,000 total cells analyzed by flow cytometry following exposure to experimental conditions in triplicate experiments.

^b ^Actual values presented as log_10 _comparison of viability between 0 h and 1 or 6 h exposure to experimental conditions (Figure 6a). Percent viability values were calculated based on the change in viability between 0 h and 1 or 6 h exposure for each experimental condition (Figure 6a).

^c ^Percentage of viable cells was not applicable due to cell growth in LB over time.

Based on the projected killing kinetics for MRSA, we determined 15 h as the minimum amount of time required for bactericidal activity for both CB07-L and BY07-L (Figure 1b). Following 15 h growth of MRSA in TSB, CFU plate enumeration resulted in an average of 10^9 ^CFU/mL viable cells (Figure 6b). Flow cytometric analysis of these broth-exposed cells showed an average of 95.8% of cells with intact membranes (Figure 7b; Table 2). Following 15 h exposure of high concentrations of MRSA to both CB07-L and BY07-L, CFU plate enumeration resulted in bactericidal activity with a 99.99% (4-log_10_) decrease in viability (Figure 6b). Flow cytometric analysis of these cells demonstrated that 3.2% and 1.7% of CB07-L- and BY07-L-exposed cells, respectively, have intact membranes (Figure 7h and 7j; Table 2). Table 2 summarizes the MRSA viability and membrane permeability demonstrating that these data indicate that membrane permeabilization occurred in MRSA during the 15 h exposure to the leachates, and thus contributed to bactericidal activity in this organism. MRSA cells were also exposed to a low pH phosphate buffer control to mimic the low pH environment of the leachates. As expected, flow cytometric analyses revealed an average of 2.6% of low pH buffer-exposed MRSA cells with intact membranes (Figure 7f; Table 2).

**Table 2:** Summary of MRSA survival and membrane permeability following incubation in TSB, water, pH 3.6 phosphate buffer, and mineral leachates

Treatment	Time (h)	Flow cytometry, percent intact membranes **(avg ± SD)**^**a**^	CFU enumeration **(% viability reduction)**^**b**^
TSB	15	96.6 (95.8 ± 2.6)	2-log_10 _Increase (n/a)^c^
			
dH_2_O	15	61.9 (78.0 ± 16.0)	1-log_10 _Decrease (90.0)
			
pH 3.6 Buffer	15	6.1 (2.6 ± 3.0)	5-log_10 _Decrease (99.999)
			
CB07-L	15	3.2 (3.3 ± 2.4)	4-log_10 _Decrease (99.99)
			
BY07-L	15	1.7 (1.7 ± 1.0)	4.5-log_10 _Decrease (99.995)

^a ^Actual percentages based on 50,000 total cells analyzed by flow cytometry following 15 h exposure to experimental condition in triplicate experiments.

^b ^Actual values presented as log_10 _comparison of viability between 0 h and 15 h exposure to experimental conditions (Figure 6b). Percent viability values were calculated based on the change in viability between 0 h and 15 h exposure for each experimental condition (Figure 6b).

^c ^Percentage of viable cells was not applicable due to cell growth in TSB over time.

**Figure 8:**
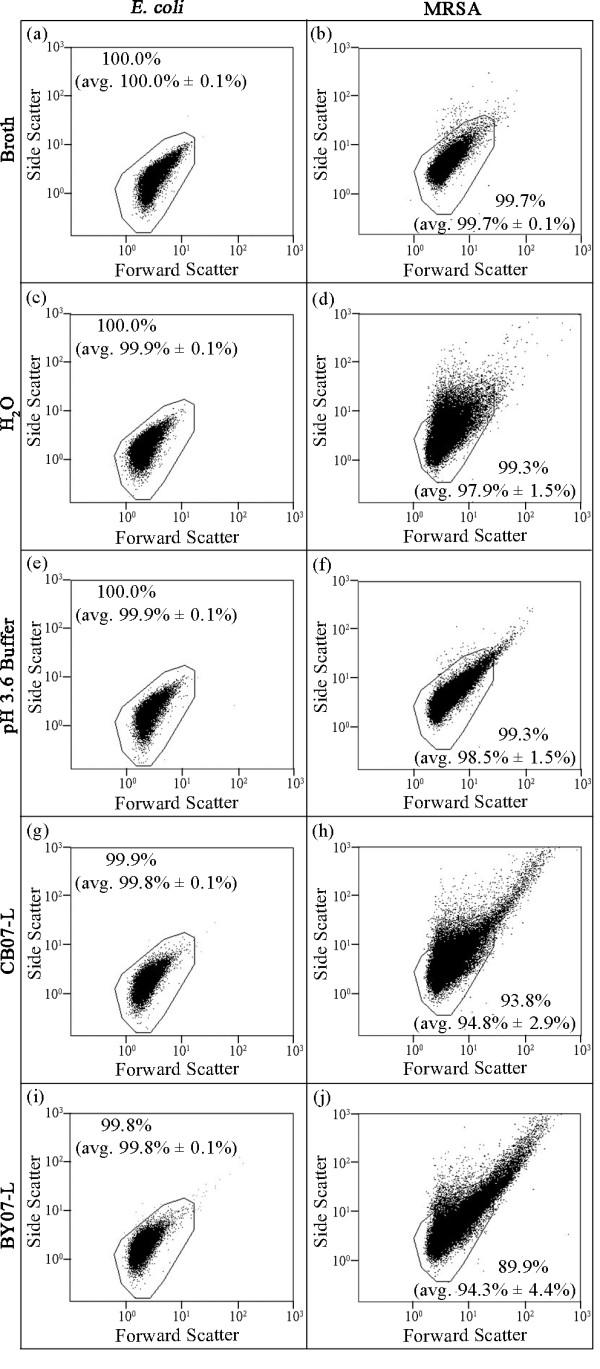
**Forward-scatter and side-scatter dot plots of *E. coli *(a, c, e, g, i) and MRSA (b, d, f, h, j) determined by flow cytometry after incubation in broth, dH_2_O, pH 3.6 buffer, CB07-L, or BY07-L**. Flow cytometric dot plot analyses and percentages were derived from a single representative experiment. Average values from at least three independent replicate experiments are reported in parentheses. Percentages denote the number of cells falling inside the gate.

While only 2.6% of MRSA cells had intact membranes following exposure to the low pH buffer, comparable to the leachate-exposed cells, several remarkable differences were observed between these two populations. First, the population profile on the forward-scatter side-scatter plots (FS - SS) of MRSA cells exposed to broth medium, dH_2_O, and the low pH buffer maintained a consistent shape, with < 2% of each population falling outside the gated area (Figures 8b,d, and 8f). In contrast, a marked spread of the population on the FS - SS plot occurred following exposure to CB07-L and BY07-L, resulting in an average number of cells of 5.2% and 5.7%, respectively, excluded by the gate (Figures 8h and 8j). These differences demonstrated that changes in cell shape and complexity occurred during exposure to the leachates, but not during exposure to TSB, dH_2_O, or the low pH buffer. These flow cytometry analyses corroborate the observed changes in MRSA cell shape seen in TEM images following exposure to the leachates (Figure 4), demonstrating that the appearance of distorted cells (Figure 5b) is characteristic of leachate exposure and not due to TEM processing. Additionally, while the MRSA cells exposed to the low pH buffer maintained the highly red-fluorescent transitional population (Figure 7f; black arrow), it did not display the lower-intensity red-fluorescent population that was characteristic of the leachate-exposed cells (Figures 7h and 7j; white arrows). These differences demonstrated that while the total number of membrane permeabilized cells was similar following MRSA exposure to the leachates and pH buffer, the respective differences in fluorescence staining patterns indicated that the mechanism of toxicity differs between the low pH environment alone and the leachates. No changes in size, shape, and complexity were observed following *E. coli *exposure to CB07-L and BY07-L, as demonstrated by 99.9% and 99.8% of cells, respectively, remaining inside the FS-SS gate (Figures 8g and 8i). Likewise, SEM and TEM images showed *E. coli *cells incubated in broth showed marked similarity in cell shape compared to those exposed to the antibacterial leachates (Figures 2 and 4).

## Discussion

The abundance of antibiotic resistant pathogens has incrementally increased since the introduction of penicillin in the 1940s. In the United States alone, more than 70% of hospital-acquired infections are antibiotic-resistant, and community-acquired exposure to antibiotic-resistant pathogens is becoming increasingly prevalent [25,26]. This problem is further exacerbated by waning research and development of novel antibacterial agents. Historically, new antibiotics were developed by recapitulating a small set of molecular scaffolds, thus allowing opportunities for further antibiotic resistance to develop [27,28]. These alarming trends highlight the urgent need to develop novel and alternative antibacterial agents.

In the past 25 years, ~70% of commercially available antibiotics have been derived from natural sources [28,29]. However, many unexplored natural resources remain promising for the discovery of new antibacterial agents. Clay minerals have been used historically for cosmetic purposes and to treat ailments of the digestive tract, but more recently have been investigated for their potential antibacterial properties [2,30]. The research presented here documents an understanding into the mechanism of action of mineral leachates, CB07-L and BY07-L, and scientifically validates the antibacterial efficacy of these clay mineral mixtures as promising new antibacterial agents. Further, an understanding of the antibacterial mechanism of action of these natural products will allow development of a chemically-derived, synthetic alternative, thus guaranteeing consistent efficacy.

Previously, we showed that the BY07 and CB07 mineral mixtures are composed of 37.3% and 21.4% smectite, respectively [5]. Smectite clays have a layered structure with an expandable interlayer and a high cation exchange capacity attributed to their overall net negative charge. In a hydrated suspension, these cations can be exchanged with ions in the external solution, provided charge balance is maintained [31]. In a low pH environment, the abundant protons saturate metal binding sites in the solution, maximizing the concentration of soluble metal ions. Consequently, metal ions become more bioavailable, and possibly more toxic, as the pH of a solution decreases [15]. Some metal ions, such as iron, copper, nickel, magnesium, manganese, and zinc, have specific biological functions as enzyme cofactors or to stabilize proteins and bacterial cell walls [15,32]. Alternatively, ions, such as aluminum, arsenic, lead, and mercury, have no biological function. Such metals can exert toxic effects by irreversibly binding sulfhydryl groups in proteins or enzyme metal binding sites [19,33]. Regardless of their function, all metals can exert toxic effects at high concentrations due to non-specific binding [15]. As a consequence, microorganisms have adapted several mechanisms, such as active transport, sequestration, and enzymatic detoxification to exclude heavy metals and regulate intracellular concentrations of essential metals [34-36]. Moreover, when metal ions are present in combination, toxicity can be magnified due to synergistic effects. For example, when lead and mercury are present together, the toxic effects are amplified 100-fold [37]. It is likely that the toxic effects of these mineral mixtures are due to the synergistic effects mediated by a combination of ions present in the solution [5]. However, further research is needed to determine which elements are mediating toxicity and their specific molecular targets.

Many groups have used EM to visualize cellular ultrastructure following exposure to silver ions [22,38,39], however, minimal literature exists on the use of EM to assess antibacterial activity and ultrastructural influences of other metal ions. Following exposure to silver ions, *E. coli *and MRSA cells exhibit condensed DNA and electron-dense granules bound to the cell envelope [22,38], in a similar manner as the CB07 and BY07 leachates (Figures 2 and 4). To our knowledge, we report the first EM ultrastructural analysis of *E. coli *and MRSA following exposure to antibacterial mineral leachates, demonstrating that neither *E. coli *nor MRSA cells lysed following exposure to the mineral leachates. Furthermore, changes in cell shape observed in MRSA cells following exposure to the leachates were not evident in *E. coli*. Condensation of the cytoplasm was observed in *E. coli*, and small (~ 10 nm), electron-dense deposits were visualized on the surface of the cells.

CB07-L and BY07-L exhibit bactericidal activity against *E. coli *and MRSA as determined by CFU enumeration. Overall, a phenomenon occurs whereby *E. coli *is rapidly killed by CB07-L and BY07-L without permeabilization to the membrane, while MRSA is killed at a slower rate with membrane permeabilization. Tables 1 and 2 summarize the membrane permeability data determined by flow cytometry and cell viability determined by CFU enumeration. Notably, following a 6 h exposure to BY07-L, *E. coli *viability decreased by 2.5 log_10 _units (99.5% decrease), while LIVE/DEAD staining determined that only 0.7% of the cell membranes were permeabilized. These data demonstrate the careful consideration needed when using LIVE/DEAD staining as a direct indicator of cellular viability.

It is known that bacterial cell walls interact strongly with metal cations, maintaining control over the type and amount of ions that gain access to the cytoplasm [40]. Gram-positive cell walls specifically have been shown to have a higher charge capacity, allowing containment of a larger volume of cations [41]. For example, it has been demonstrated that *Bacillus subtilis *binds 28 to 33 times more Cu^2+ ^than *E. coli *[41]. As a potent metal ion chelator, the thick peptidoglycan layer present in *S. aureus *likely contributes to the delayed toxicity seen with MRSA exposure to the leachates. Moreover, while these ions are trapped in the peptidoglycan, they can propagate oxidative damage to the membrane [42]. Because *E. coli *is killed without loss of membrane integrity, it is possible that membrane permeabilization observed in MRSA cells is not the principle mechanism of action, but rather a secondary consequence due to the slow passage through the thick peptidoglycan.

MRSA exposure to the leachates resulted in a distinct and reproducible fluorescence staining pattern as observed with flow cytometry. Interestingly, the dot plots of leachate-exposed MRSA cells show two red-fluorescent populations. The first population moved in a distinct curve-shaped manner, transitioning from higher red fluorescence intensity to lower red fluorescence intensity (Figures 7h and 7j; black arrow). This feature is likely due to intermediate states, characterized by different concentrations of SYTO9 and PI dyes within the cells [24]. The second population, although still red, occurred at much lower fluorescence intensity (Figures 7h and 7j; white arrow). This phenomenon could possibly be due to an overall lower abundance of nucleic acids in these cells. Alternatively, due to FRET, PI fluoresces with greater intensity when in the presence of SYTO9 and at a lower intensity when present alone [20]. Therefore, the separation of these two populations may be due to a greater abundance of SYTO9 in the upper population and a decreased abundance of SYTO9 in the lower population.

Natural sources have historically played an important role in the discovery of novel antibacterial agents [29]. CB07 and BY07 mineral mixtures and their leachate derivatives could offer an additional complementary treatment option against topical bacterial infections. However, efficacy of these minerals can vary widely despite having a common source. It is therefore essential to characterize their specific antibacterial mechanism of action in order to improve quality control, guarantee consistent efficacy, and maximize their performance as an antibacterial agent.

## Conclusions

In summary, these data suggest that the mineral leachate antibacterial killing activity differs for Gram-positive and Gram-negative organisms and have guided us in our understanding of the leachate antibacterial mechanism of action. Upon antibacterial mineral leachate exposure, structural integrity is retained, however, compromised membrane integrity accounts for bactericidal activity in Gram-positive, but not in Gram-negative cells.

## Competing interests

The authors declare that they have no competing interests.

## Authors' contributions

CCO performed the antimicrobial susceptibility experiments, developed protocols for and performed the MRSA TEM, SEM, and *Bac*Light LIVE/DEAD flow cytometry experiments, analyzed the MRSA TEM and SEM data, interpreted the flow cytometry data, and wrote and edited the manuscript. TMC participated in the initial design of the study, developed protocols for and performed the initial *E. coli *TEM and *Bac*Light LIVE/DEAD staining experiments, and edited the manuscript. MRH performed the *E. coli *TEM, SEM, and *Bac*Light LIVE/DEAD flow cytometry experiments, analyzed the *E. coli *TEM and SEM data, provided technical support for the flow cytometry experiments, interpreted the flow cytometry data, and edited the manusript. SEH conceived of the study, participated in the design and coordination of the experiments, analyzed the data, and helped to write and edit the manuscript. All authors read and approved the final manuscript.
